# Supplementary material for: Microwave-transparent metallic metamaterials for autonomous driving safety
Source: Nat Commun. 2024 May 28;15:4516. doi: 10.1038/s41467-024-49001-w (PMC11130274; doi:10.1038/s41467-024-49001-w)
Supplement: Supplementary file 1 — Supplementary Information [file 41467_2024_49001_MOESM1_ESM.pdf]

Microwave-Transparent Metallic Metamaterials for Autonomous Driving Safety

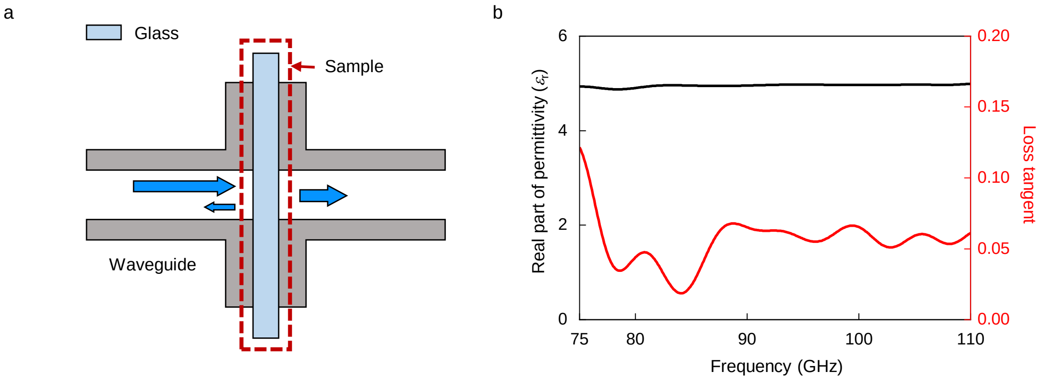

Supplementary Fig. 1| Complex permittivity dispersion of a glass cover. a, Schematic of a measurement setup for obtaining complex permittivity dispersions at microwave frequencies. The permittivity dispersion of a sample is determined through a retrieval process with transmitted and reflected signals. **b,** Measured real part of permittivity, ε_r (black line) and loss tangent (red line) of an Eagle-XG glass cover within the W-band (75–110 GHz).

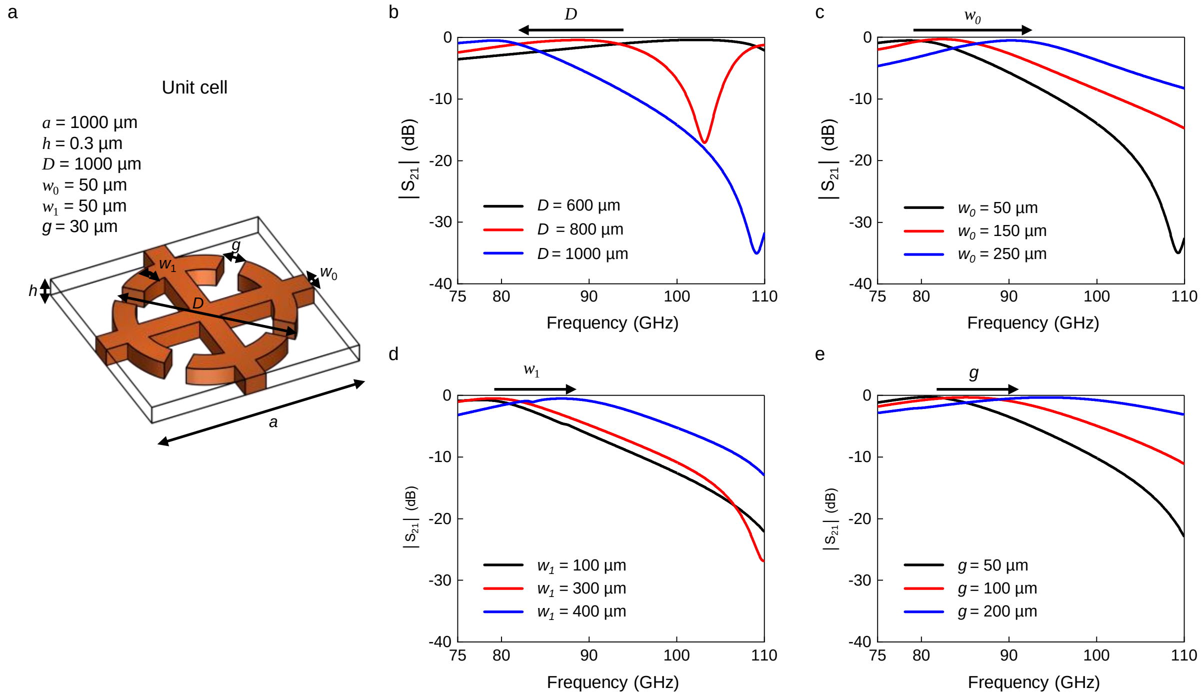

Supplementary Fig. 2| Primary structural parameters of a metamaterial design. a, Schematic illustrating the unit cell of a metamaterial array. For all the simulations, *a* and *h* are fixed at 1000 µm and 0.3 µm, respectively. **b**–**e,** Simulated S_{21} spectra with individually increasing the parameter of (**b**) *D*, (**c**) w_0, (**d**) w_1, and (**e**) *g*, while the other parameters are fixed.

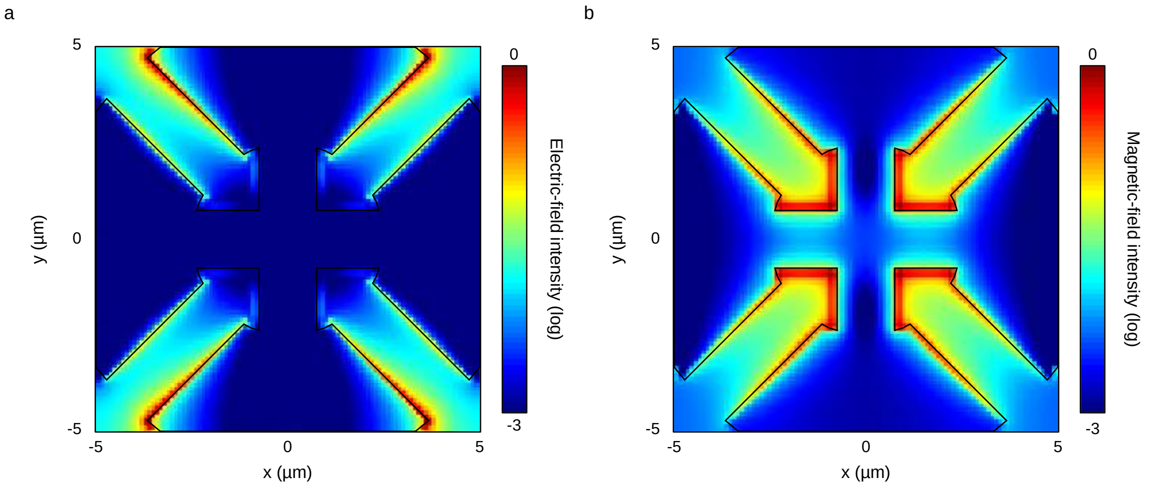

Supplementary Fig. 3| Electromagnetic response of the metamaterials. a, Electric- and **b,** magnetic-field intensity profiles at the cross-section of the Meta III, in response to an x-polarised microwave signal at 91.3 GHz.

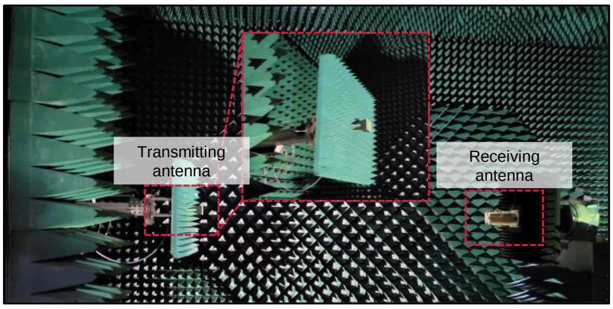

Supplementary Fig. 4| Measurement setup for microwave transmission spectrum. Photograph of a measurement setup for obtaining microwave transmission spectrum. Measurements on fabricated samples are conducted using a vector network analyser in an anechoic chamber. The obtained results are shown in Fig. 1c, 3b and inset of 3e**.**

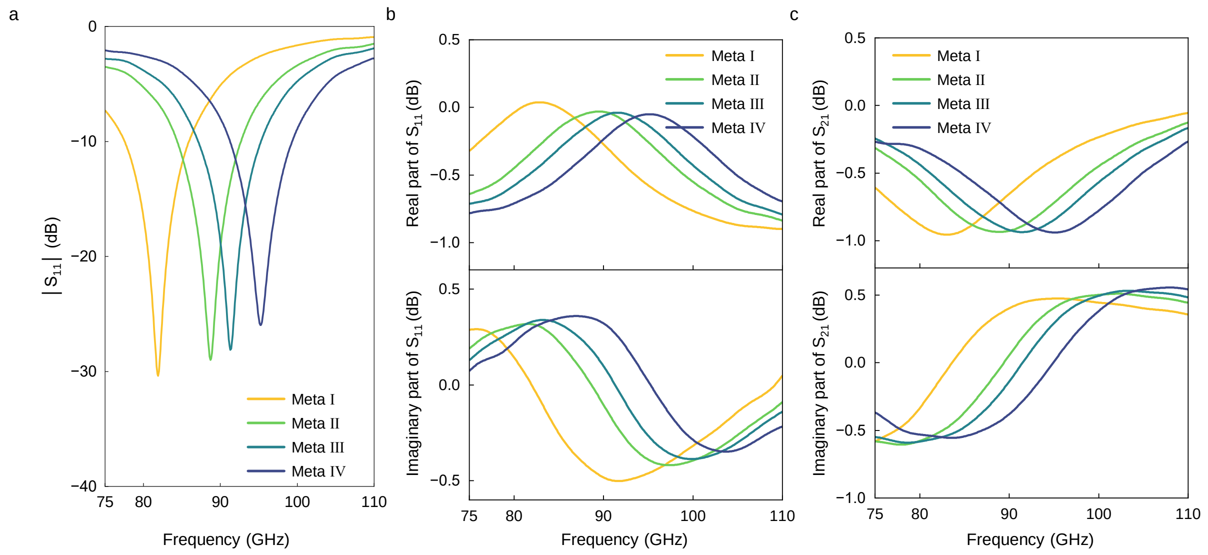

Supplementary Fig. 5| Measured complex scattering parameters of the fabricated metamaterials. a, Measured S_{11} spectra of the designed microwave-transparent metamaterials in Fig. 3b. **b,** Real and imaginary parts of the measured complex S_{11} spectra of the four fabricated samples (Meta I–IV). **c,** Real and imaginary parts of the measured complex S_{21} spectra of the four fabricated samples (Meta I–IV).

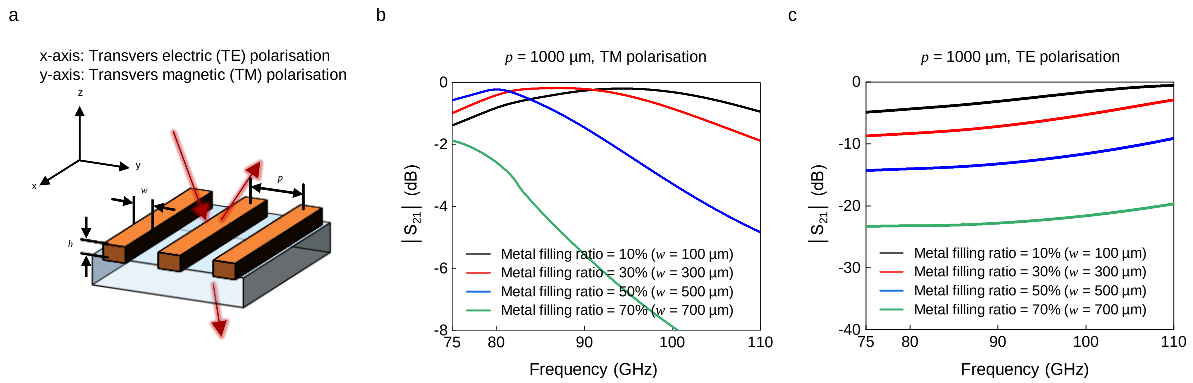

Supplementary Fig. 6| The microwave-transmittance (S_{21}) spectra of 1D wire grid structure. a, Schematic illustrating 1D wire grid arrays, characterised by structural parameters of *p*, *w* and *h*. For all simulations, *p* and *h* are fixed at 1000 mm and 0.3 mm, respectively. **b, c,** Simulated S_{21} spectra with increasing the parameter of *w* for (**b**) transverse magnetic (TM) polarisation and (**c**) transverse electric (TE) polarisation, while the other parameters are fixed.

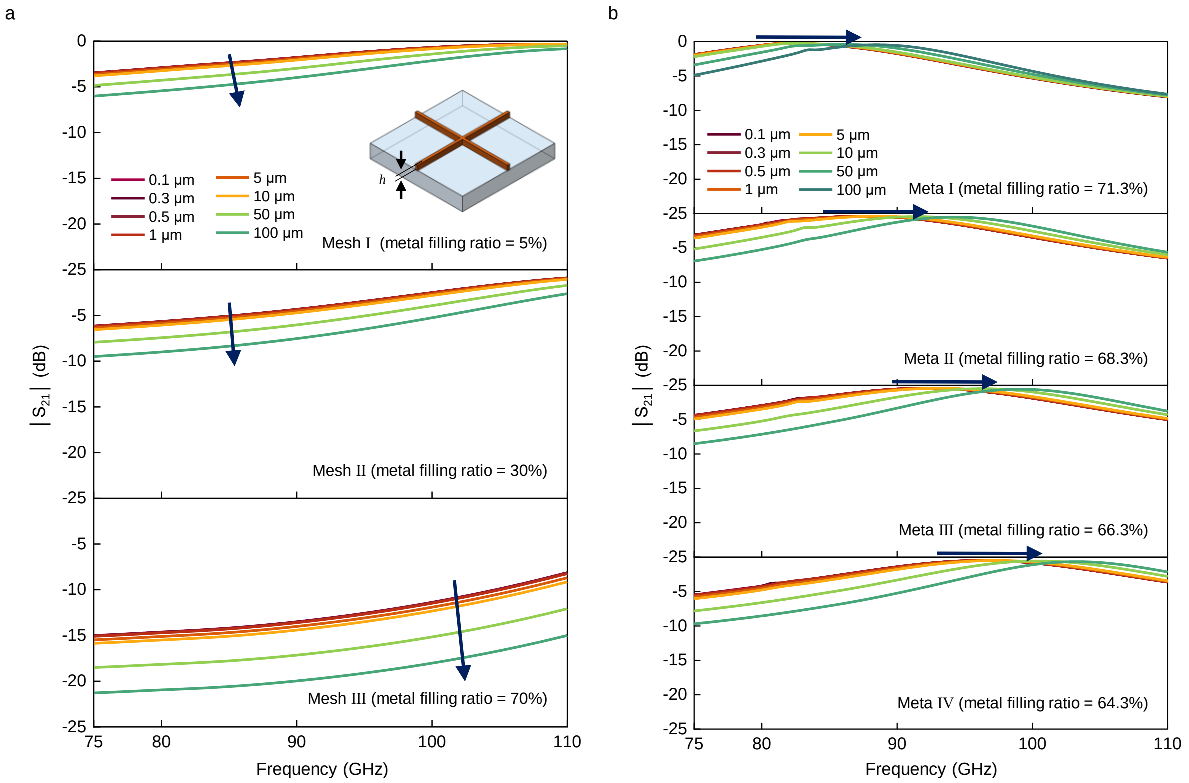

Supplementary Fig. 7| Transmission (S_{21}) spectra as a function of metal thickness. a, Simulated W band transmission spectra of (**a**) meshes and (**b**) metamaterials with the thickness (*h*) of 0.1, 0.3, 0.5, 1, 5, 10, 50 and 100 μm, respectively. Metal filling ratios are 5%, 30% and 70% for Mesh of Mesh I–III, and 71.3%, 68.3%, 66.3% and 64.3% for Meta I–IV. Spectra for *h* = 0.3 μm are the same data as displayed in Fig. 3b.

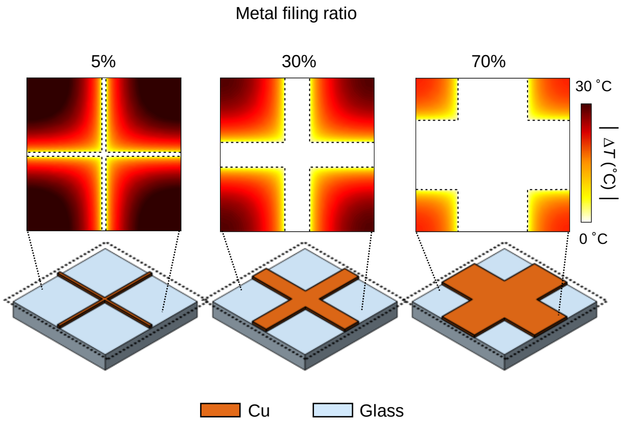

Supplementary Fig. 8| Heat transfer simulation on mesh arrays. Simulated temperature distribution on a glass cover (Eagle-XG) incorporating mesh arrays. Metal filling ratios are 5%, 30% and 70% (Mesh I–III) for mesh arrays. The variation in colour within the area corresponds to the relative temperature gradients, with white representing the highest temperature and darker colour indicating a decrease in temperature.

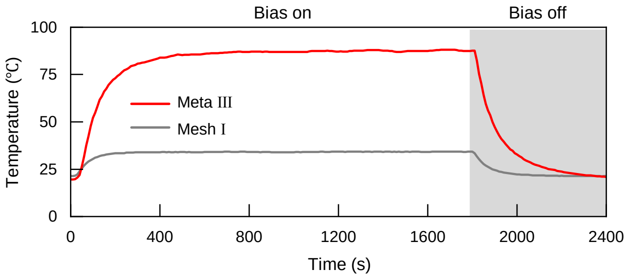

Supplementary Fig. 9| Heating test of microwave-transparent heaters. Temporal changes in temperature of Meta III and Mesh I under an input voltage of 1.6 V.

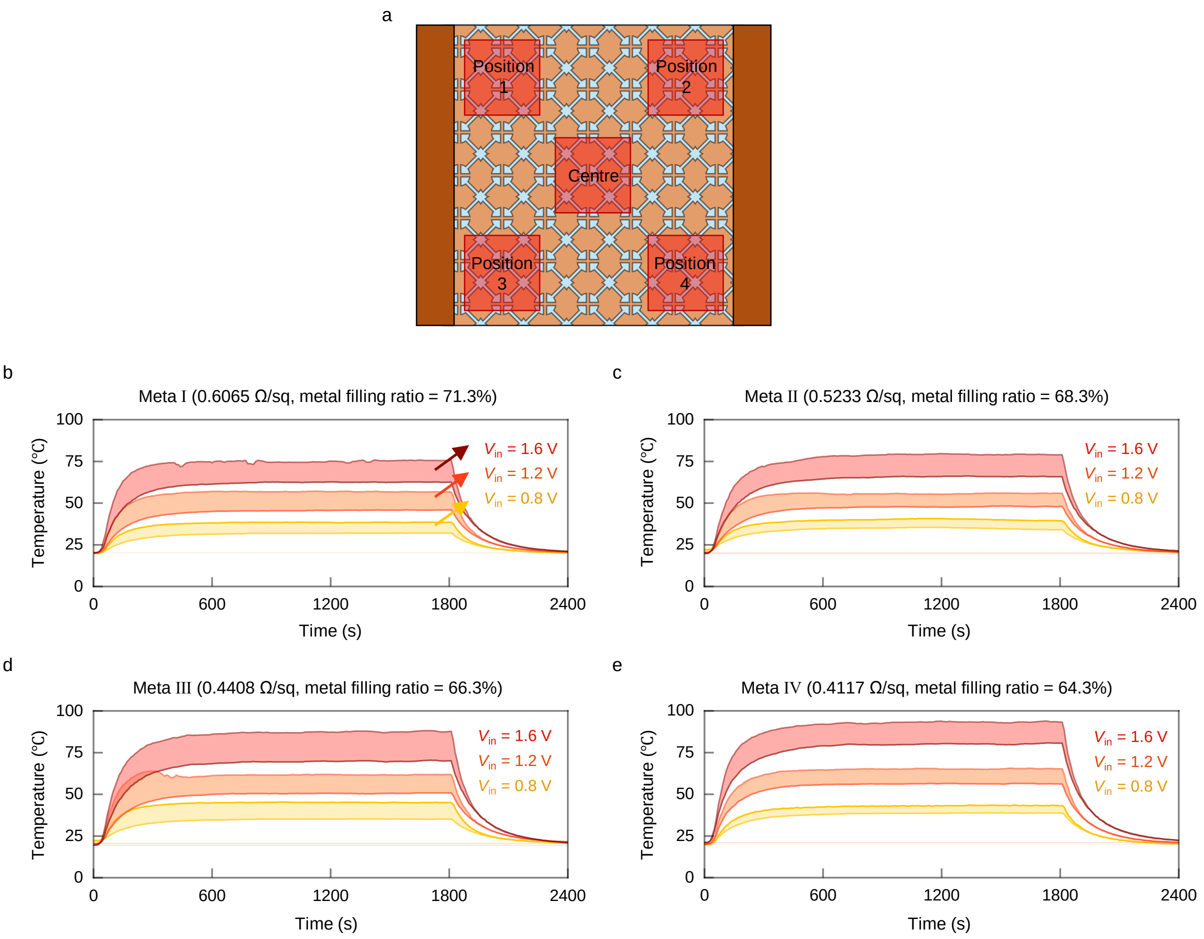

Supplementary Fig. 10| Heating tests of metamaterial-based samples. a, Schematic illustrating temperature uniformity tests for which temperatures are recorded at designated positions (Position 1–5). Thermocouples were contacted on the centre and four edges of the glass cover of each sample. **b–e,** Measured temperature variations for (**b**) Meta I, (**c**) Meta II, (**d**) Meta III and (**e**) Meta IV. The heating tests were conducted with discrete input voltages of 0.8, 1.2, and 1.6 V. The upper and lower limits of each band represent the highest (on the centre) and lowest (on the edges) temperatures, respectively. Noteworthily, the temperature variations are largely attributed to the asymmetric electrode configuration. Properly designed electrodes could help minimize temperature differences between the centre and edges.

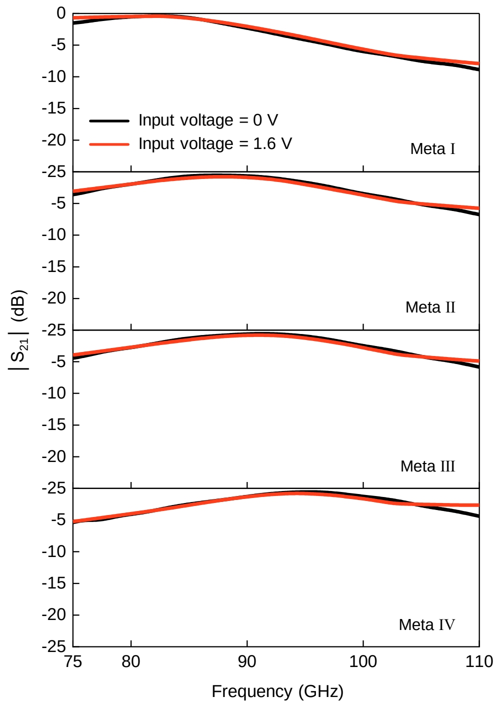

Supplementary Fig. 11| Transmission (S_{21}) spectra of metamaterials with temperature variations. Measured transmission (S_{21}) spectra of the fabricated samples (Meta I–IV) at input voltages of 0 and 1.6 V, corresponding to the range of temperatures between 76–94 °C for Meta I–IV, respectively.

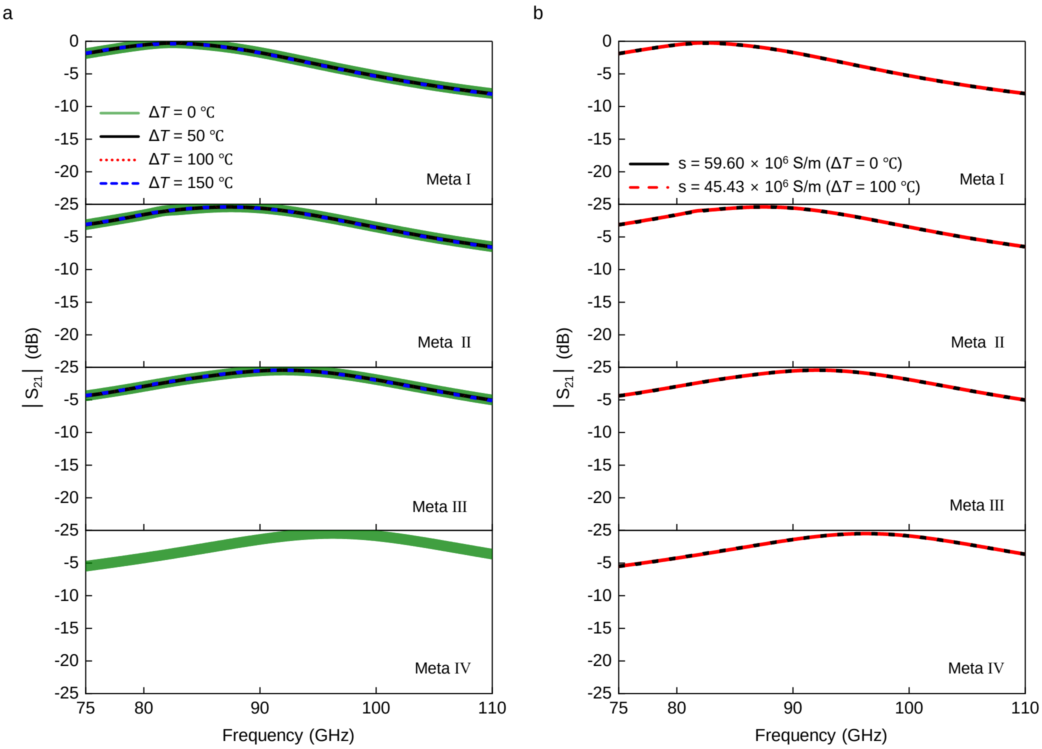

Supplementary Fig. 12| S_{21} spectra of the metamaterials at different temperatures. a, Simulated microwave transmittance (S_{21}) spectra of the metamaterials (Meta I–IV), by adjusting their structural parameters (D, w_0, w_1 and g) in consideration of temperature increases (ΔT) at 0 °C, 50 °C, 100 °C and 150 °C. **b,** Simulated microwave transmittance (S_{21}) spectra of the metamaterials (Meta I–IV) with the electric conductivity of 59.60×10^6 S/m and 45.43×10^6 S/m, respectively.

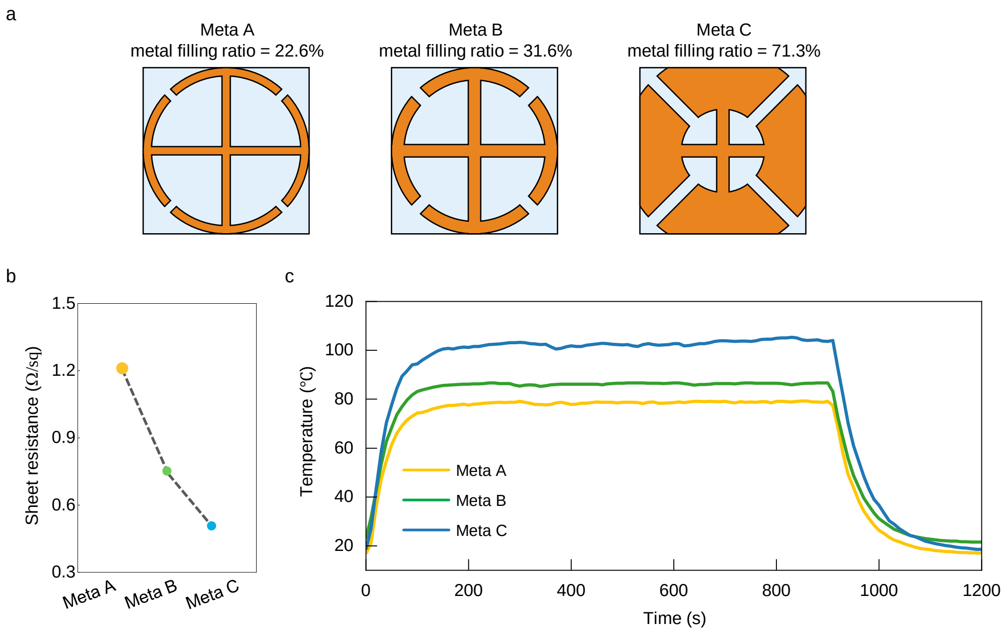

Supplementary Fig. 13| Heating tests of metamaterial-based samples with various metal filling ratios. a, Design schematics of Meta A–C with metal filling ratio of 22.6%, 31.6% and 71.8%, respectively. The key parameters of the fabricated metamaterials are listed in Supplementary Table S1. h = 0.4 mm for these samples. **b,** Measured sheet resistance values of the fabricated samples on glass substrates. **c,** Temporal changes in temperature of the fabricated samples under an input voltage of 2.4 V.

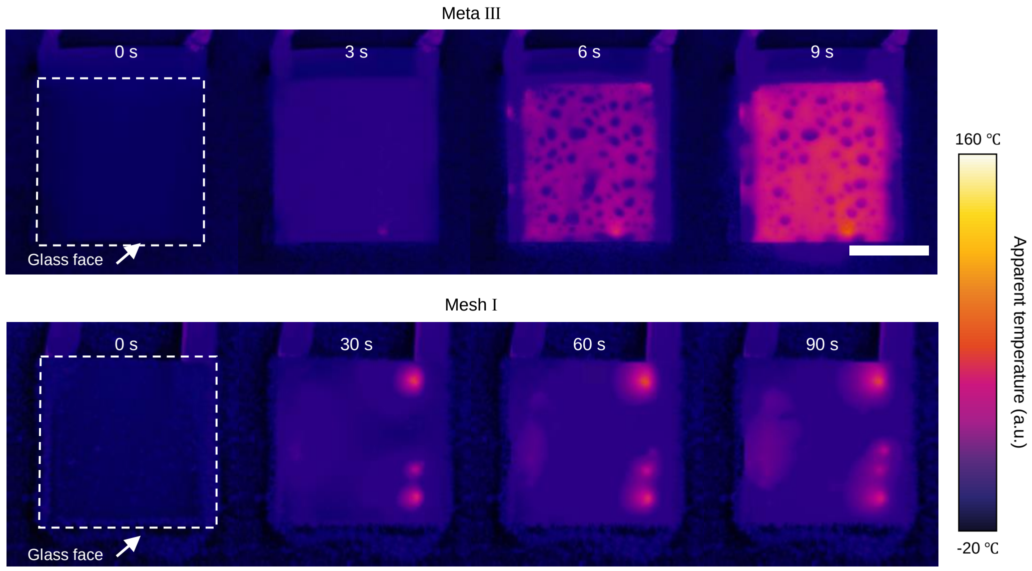

Supplementary Fig. 14| Thermographic images in defrosting experiments. Temporal changes of thermographic images of the Meta III (top) and Mesh I (bottom) samples under an input voltage of 4.5 V during the defrosting test in Fig. 4c. Scale bar, 2.5 cm.

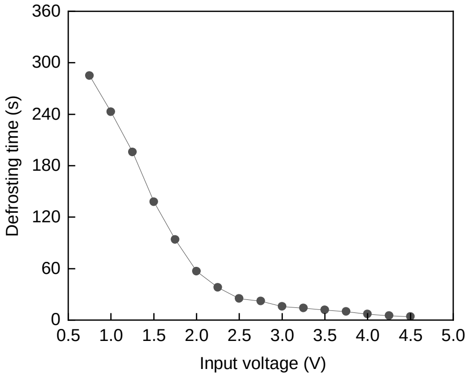

Supplementary Fig. 15| Defrosting tests of the metamaterial-based transparent heater. Measured defrosting time of the metamaterial-based (Meta IV) sample while the input voltages are varied from 0.75 to 4.5 V in increments of 0.25 V. The sample was placed on a Peltier module set to sustain a temperature of -20 °C.

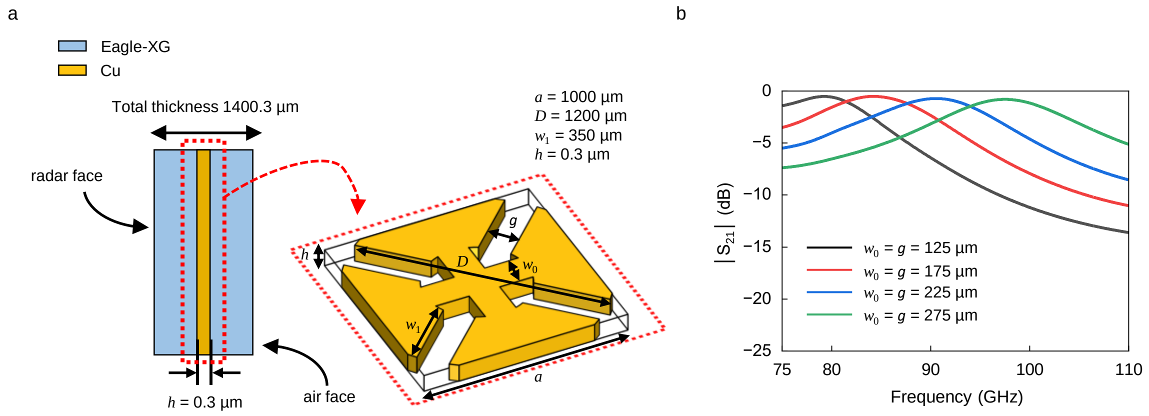

Supplementary Fig. 16| Microwave-transparent heaters with a protective cover and substrate. a, Schematic of a metamaterial embedded glass substrate, illustrating its structural parameters of a, D, w_0, w_1, h and g values. **b,** Simulated transmission (S_{21}) spectra (75–110 GHz) of metamaterial embedded glass substrates while w_0 and g values are varied. For all the simulations, a = 1000 µm, D = 1200 µm, w_1 = 350 µm and h = 0.3 µm. Their metal (Cu) filling ratios are within 40–70%. These simulations support near-unity transmission of embedded metamaterial designs with high metal filling ratios.

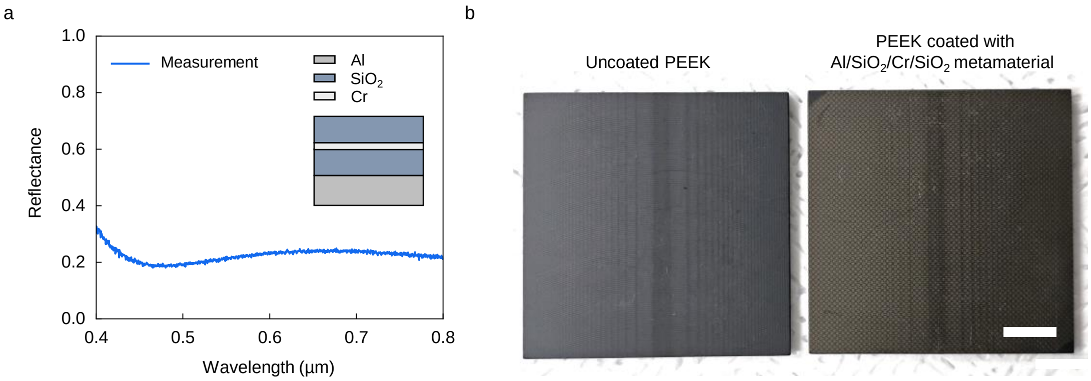

Supplementary Fig. 17| Concealment for microwave-transparent heaters. a, Measured reflectance spectrum (0.4–0.8 μm) of a SiO_2 (80 nm)/Cr (10 nm)/SiO_2 (80 nm)/Al (100 nm) multilayer. **b,** Visible camera images of black polyether ether ketone (PEEK) substrates without (left) and with a metamaterial design (right). The metamaterial design is identical to Meta 1 in Fig. 3b, consisting of the SiO_2/Cr/SiO_2/Al multilayer configuration. Its filling ratio is 71.3%. Scale bar, 1 cm.

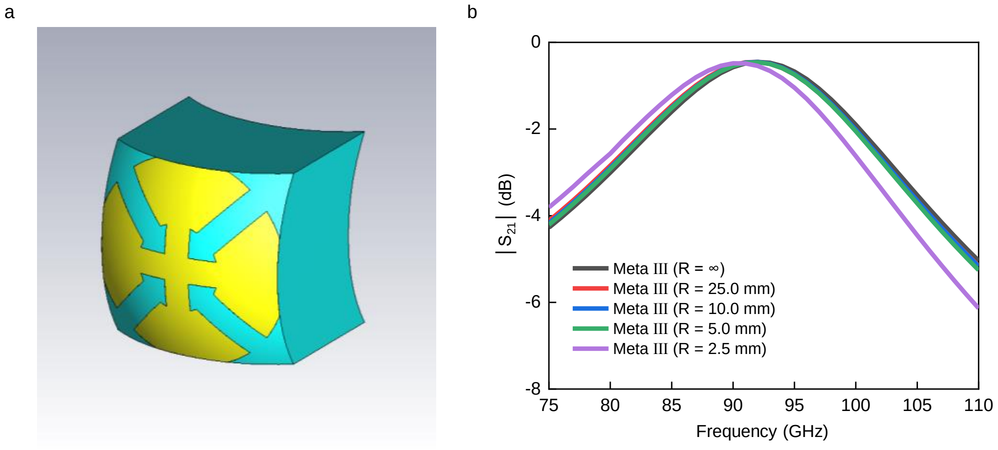

Supplementary Fig. 18| Metamaterial on a spherical curvature. a, Schematic illustration of a unit metamaterial pattern on a spherically curved substrate defined by radius of curvature R. Structural parameters (a, D, w_0, w_1, g) are same as the meta III in the manuscript. **b,** Simulated S_{21} spectra as a function of the R. Black line is the S_{21} spectra from metamaterials on the planar substrate, which is same data displayed in Fig 3b.

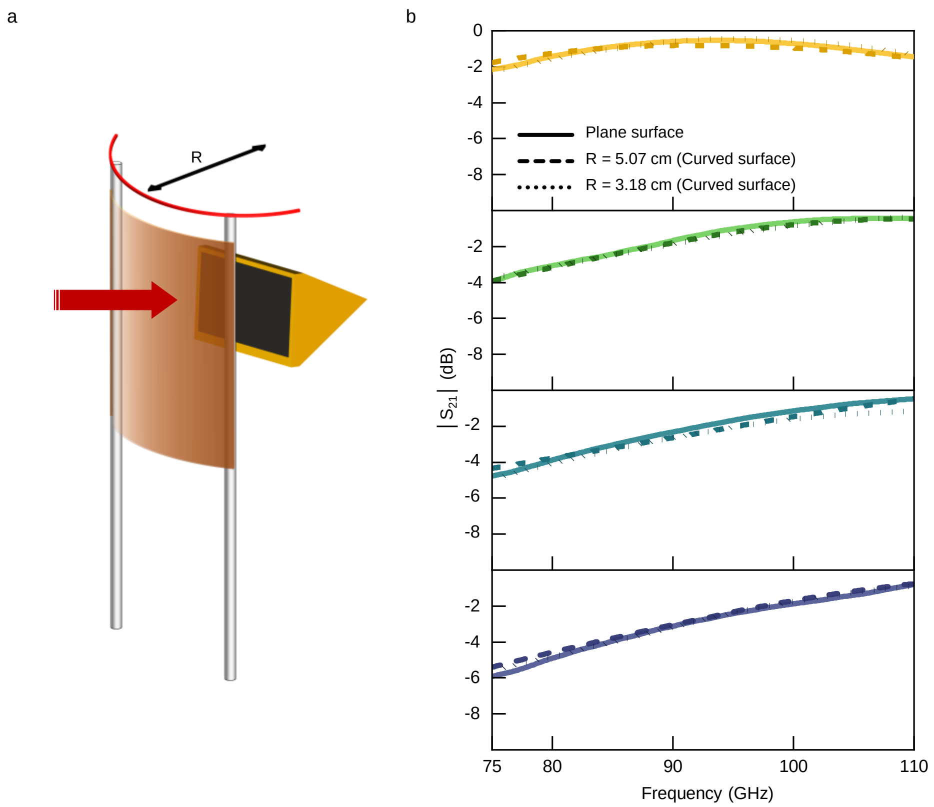

Supplementary Fig. 19| Transmission measurement of metamaterials on curved flexible substrates. a, Schematic illustration of transmission measurement setup to apply defined curvature on a flexible polymer substrate. **b,** Measured transmission (S_{21}) spectra of four fabricated metamaterials (Meta I–IV) on polymer substrates. Solid lines indicate S_{21} spectra on planar surfaces for comparison.

Supplementary Table 1| Parameters of microwave-transparent metamaterials. Parameters of the designed metamaterials shown in Fig. 2b.

	(i)	(ii)	(iii)	(iv)	(v)	(vi)
a (μm)	1000	1000	1000	1000	1000	1000
D (μm)	1000	1000	800	900	1000	1200
w_0 (μm)	50	75	75	75	75	75
w_1 (μm)	50	75	75	250	300	350
g (μm)	50	75	75	75	75	75
Metal filing ratio (%)	22.6	31.6	41.9	50.3	65.3	71.0
Maximum transmission frequency (GHz)	79.8	83.0	91.4	94.5	88.8	77.7

Supplementary Table 2| Parameters of microwave-transparent metamaterials. Parameters of the designed metamaterials shown in Fig. 2d.

	Metamaterial			Mesh-array		
	Yellow symbol	Green symbol	Blue symbol	Yellow symbol	Green symbol	Blue symbol
D (μm)	1000	1100	1200	-	-	-
w_0 (μm)	50	75	75	50	150	225
w_1 (μm)	50	150	350	-	-	-
g (μm)	50	50	75	-	-	-
Metal filing ratio (%)	22.6	46.4	71.0	19	51	69.8
Maximum transmission frequency (GHz)	79.8	75	77.7			

Supplementary Table 3| Parameters of metamaterials with high metal filling ratios. Parameters of the microwave-transparent metamaterials shown in Fig. 3b.

	Meta I	Meta II	Meta III	Meta IV
a (μm)	1000	1000	1000	1000
D (μm)	1200	1200	1200	1200
w_0 (μm)	100	125	150	175
w_1 (μm)	350	350	350	350
g (μm)	100	125	150	175
Metal filing ratio (%)	71.3	68.3	66.3	64.3
Maximum transmission frequency (GHz)	82.5	87.4	91.3	95.3

Supplementary Table 4| Previously reported transparent heaters. Composition material, performance, and working wavelength of previously reported transparent heaters. Note that all the data for comparison are extracted from visibly transparent heaters. To our best knowledge, our work presents the first report of microwave-transparent heaters.

Reference	Structure	Material	Transmittance (%)	Sheet resistance (Ω/sq)	Heating temperature (℃)
[12]	Multilayer	FTO/AZO	89.0 at 550 nm	36.7	71.8 °C at 12 V
[13]	Multilayer	ITO/Ag/ITO	88.2 at 550 nm	3	110.8 °C at 5 V
[14]	Multilayer	ZTO/Ag/ZTO	86.0 at 550 nm	7	100 °C at 6 V
[15]	Multilayer	FTO/Ag/FTO	83.0 at 550 nm	8.03	84.37 °C at 6 V
[16]	Nanowire	Ag/PDMS	93.0 at 550 nm	30	26.43 °C at 5 V
[17]	Nanowire	Ag	90.0 at 420–800 nm	4.82	343.5 °C at 9 V
[18]	Nanowire	Ag/PET	95.0 at 550 nm	15	45.9 °C at 5 V
[19]	Nanowire	Ag/PET	85.0 at 550 nm	0.5	238.71 °C at 4.5 V
[20]	Nanowire	Ag	89.0 at 550 nm	0.195	199.72 °C at 2.5 V
[36]	Nanowire	EGaIn	90.1 at 550 nm	1.7	243.27 °C at 2.5 V
[37]	Nanowire	Ag	77.0 at 550 nm	13.3	102 °C at 6 V
[39]	Mesh	Nano-RGO/ Ag NWs	88.4 at 550 nm	17.5	247.2 °C at 14 V
[40]	Hybrid film	Cu NWs/PET	61.4 at 550 nm	9.4	81.2 °C at 6 V
[41]	Hybrid film	ZnO-Ag NWs	82.5 at 400–800 nm	10	309.14 °C at 12 V
[42]	Hybrid film	Cu	83.5 at 550 nm	13.6	186.9 °C at 6 V
[43]	Hybrid film	Ag/PDDA/GO	91.0 at 550 nm	11	37.15 °C at 12 V
[44]	Hybrid film	CuZr-Ag	91.1 at 550 nm	3	15.7 °C at 0.95 V
