# Supplementary material for: Microwave-transparent metallic metamaterials for autonomous driving safety
Source: Nat Commun. 2024 May 28;15:4516. doi: 10.1038/s41467-024-49001-w (PMC11130274; doi:10.1038/s41467-024-49001-w)
Supplement: Supplementary file 3 — Description of Additional Supplementary Files [file 41467_2024_49001_MOESM3_ESM.docx]

**Description of Additional Supplementary Files**

**Supplementary Movie 1:**

Meta III and Mesh I were selected as a transparent heater and its reference sample, respectively. Prior to the defrosting experiments, the samples were subject to an environment with a temperature of -20 ℃ for 140 min. This movie contains the four videos of visible and thermographic records for Meta III and Mesh I throughout the defrosting tests. The captured images were used for Fig. 4c and Supplementary Fig. 14.
